# Alternative Concepts for Extruded Power Cable Insulation: from Thermosets to Thermoplastics

**DOI:** 10.1002/adma.202313508

**Published:** 2024-04-12

**Authors:** Amir Masoud Pourrahimi, Massimiliano Mauri, Silvia D'Auria, Roberta Pinalli, Christian Müller

**Affiliations:** ^1^ Department of Chemistry and Chemical Engineering Chalmers University of Technology Göteborg 41296 Sweden; ^2^ Department of Materials Engineering Nexans Norway AS Knivsøveien 70 Berg i Østfold 1788 Norway; ^3^ Department of Chemistry Life Sciences and Environmental Sustainability University of Parma Parma 43124 Italy

**Keywords:** click chemistry type curing, covalent and non‐covalent adaptable networks, crosslinked polyethylene (XLPE), high‐voltage direct current (HVDC) power cable, polypropylene‐based thermoplastic insulation

## Abstract

The most common type of insulation of extruded high‐voltage power cables is composed of low‐density polyethylene (LDPE), which must be crosslinked to adjust its thermomechanical properties. A major drawback is the need for hazardous curing agents and the release of harmful curing byproducts during cable production, while the thermoset nature complicates reprocessing of the insulation material. This perspective explores recent progress in the development of alternative concepts that allow to avoid byproducts through either click chemistry type curing of polyethylene‐based copolymers or the use of polyolefin blends or copolymers, which entirely removes the need for crosslinking. Moreover, polypropylene‐based thermoplastic formulations enable the design of insulation materials that can withstand higher cable operating temperatures and facilitate reprocessing by remelting once the cable reaches the end of its lifetime. Finally, polyethylene‐based covalent and non‐covalent adaptable networks are explored, which may allow to combine the advantages of thermoset and thermoplastic insulation materials in terms of thermomechanical properties and reprocessability.

## Graphical abstract

Crosslinked polyethylene is the most widely used insulation material for extruded high‐voltage power cables. This perspective explores alternative concepts such as click chemistry type curing, polyolefin blends and adaptable networks, which avoid hazardous crosslinking agents and the release of harmful byproducts. Moreover, insulation materials that remain thermoplastic may ease reprocessing once the cable has reached the end of its lifetime.

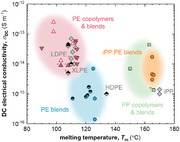

## 1 Introduction

Worldwide, we currently witness an accelerating shift from fossil and nuclear fuels to renewable sources of energy to reduce greenhouse gas emissions and their effect on global warming. These changes have influenced the development of power transmission systems. Since the beginning of electrification toward the end of the 19^th^ century, high‐voltage alternating current (HVAC) systems have dominated electric power transmission. This is mainly due to the low installation cost and low transmission losses that HVAC systems offer when transmitting power over short distances, typically not more than tens of kilometers. Fossil fuel power plants tend to be located close to urban areas, which therefore favor HVAC technology. In contrast, renewable sources of energy such as solar, wind, and hydropower are usually most abundant far away from urban areas, which results in high transmission losses if HVAC technology were to be used. High‐voltage direct current (HVDC) technology, on the other hand, allows efficient energy transport over much longer distances of hundreds or even thousands of kilometers with significantly lower transmission losses, facilitating the seamless integration of renewable sources of energy into electricity grids.^[^ ^1^ ^]^

**Figure 1:**
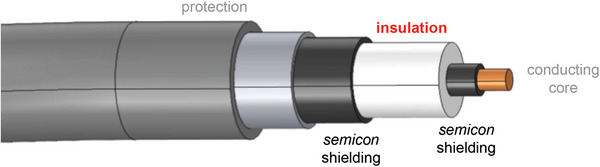
Schematic of a HVDC cable, consisting of a conducting core that is surrounded by semicon shielding, insulation and (several) protection layers, to be buried underground or laid on the bottom of the sea. Figure reproduced with permission from ref. [5]. Copyright 2019, Wiley.

HVDC transmission lines are likely to traverse large bodies of water or densely populated areas and therefore must be submerged or buried underground. Hence, an insulation layer is required that surrounds the conducting core. Among the different components of an HVDC cable (see **Figure** **1**), the insulation layer plays a central role since its ability to withstand electrical and thermomechanical stresses ultimately determines the transmission voltage *U*.^[^ ^2^ ^]^ Since the performance of an HVDC cable is given by the power *P*  =  *U* · *I* that can be transferred,^[^ ^3^ ^]^ it is desirable to maximize the voltage (an increase in the current *I* would lead to additional losses through Joule heating).^[^ ^4^ ^]^ An early type of insulation, which is still widely used today and allows a voltage as high as 600 kV and power of 2–3 GW, is based on paper impregnated with an oil (“oil‐filled” insulation) or a highly viscous compound (“mass‐impregnated” insulation).^[^ ^4^ ^]^ The most advanced type of HVDC technology, however, uses extruded polymer‐based insulation, which can withstand higher operating temperatures (70–90 °C), is easier to install/repair and today enables a record voltage of up to 640 kV and power of up to 3 GW.^[^ ^1^ ^]^ The most widely used extruded insulation is composed of low‐density polyethylene (LDPE), which is eventually crosslinked resulting in crosslinked polyethylene (XLPE).

**Figure 2:**
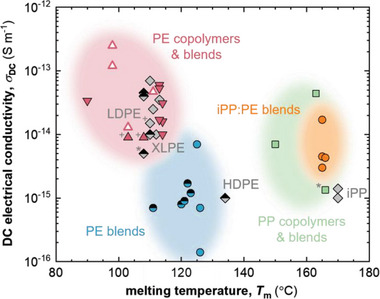
Literature values of DC electrical conductivity *σ*_*DC*_ at 70 °C (closed symbols: leakage current measurements at 30 kV mm^−1^; open symbols: broadband dielectric spectroscopy (BDS) at 10 V mm^−1^) versus highest peak melting temperature *T_m_* of LDPE,^[^ ^18^, ^19^, ^20^, ^21^, ^22^ ^]^ XLPE (crosslinked LDPE),^[^ ^18^, ^20^, ^22^, ^23^ ^]^ crosslinked HDPE^[^ ^18^ ^]^ and iPP (grey diamonds: thermoplastic; grey/black diamonds: crosslinked with DCP),^[^ ^21^, ^24^ ^]^ PE copolymers and blends that underwent click chemistry type crosslinking (upward red triangles)^[^ ^19^, ^25^, ^26^ ^]^ and ionomers (downward red triangles),^[^ ^22^ ^]^ PE blends comprising LDPE and HDPE (blue circles: thermoplastic; blue/black circles: crosslinked with DCP),^[^ ^18^, ^27^ ^]^ PP copolymers and blends (green squares)^[^ ^20^, ^23^, ^26^ ^]^ and iPP:PE blends (orange circles);^[^ ^21^, ^24^ ^] *^80 °C and 8 kV mm^−1^ and ^+^70 °C and 20 kV mm^−1^.

To understand the strategies that are available to further enhance the transmission voltage (i.e., to lower transmission losses), the heat generated by the conducting core must be considered. The insulation will experience a temperature gradient, where the region closest to the conducting core is hottest and reaches a maximum temperature *T_max_* that should not exceed the highest temperature that the insulation material can withstand. *T_max_* is related to the transmission voltage according to^[^ ^6^ ^]^:

(1) $$U^{2}\propto \int_{T_{0}}^{T_{\max}}\frac{\kappa }{\sigma_{DC}\left(T\right)}dT$$

where *κ* and *σ*_*DC*_(*T*) are the thermal and direct‐current (DC) electrical conductivity of the insulation material (*κ* and *σ*_*DC*_(*T*) are here assumed to be temperature and electric‐field independent, respectively), and *T*_0_ is the temperature of the cable surroundings. Equation 1 suggests two strategies that –besides changing the dimensions of the cable– may allow to enhance *U* and hence the transmitted power: (1) a reduction in *σ*_*DC*_ by using a more insulating material, and (2) an increase in *T_max_* by selecting an insulation material that can withstand higher temperatures. To reduce the electrical conductivity of the insulation material, different types of additives have been explored, including aromatic molecules such as anthracene,^[^ ^7^ ^]^ 4,4′‐dihydroxy benzophenone^[^ ^8^ ^]^ and benzoic acid derivatives,^[^ ^9^, ^10^, ^11^ ^]^ organic semiconductors such as C_60_,^[^ ^12^ ^]^ a dithieno‐indacenodithiophene derivative^[^ ^13^ ^]^ and poly(3‐hexylthiophene) (P3HT),^[^ ^14^ ^]^ metal oxide nanoparticles (e.g., Al_2_O_3_, ZnO, MgO),^[^ ^15^, ^16^ ^]^ and graphene oxide,^[^ ^17^ ^]^ which are not the focus of this review. We will instead focus on recent attempts to improve *T_max_* with the peak melting temperature $T_{m}^{LDPE}$ ≈110 °C of LDPE as a reference point, which is commonly used as the base material for extruded HVDC insulation. In some cases, materials that have a higher melting temperature *T_m_* and thus offer a higher *T_max_* also feature a lower *σ*_*DC*_ (**Figure** **2**; note that *T_max_* < *T_m_*), as will be discussed below. At the same time, the thermal conductivity *κ* ≈0.3 to 0.4 W m^−1^ K^−1^ of LDPE should be maintained to not complicate heat management of the cable (a reduction in *κ* would require a thinner insulation layer).

**Figure 3:**
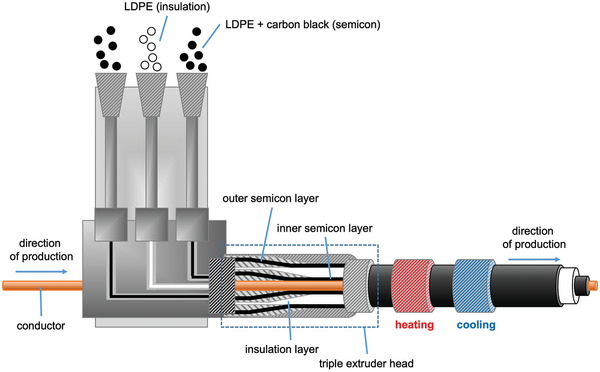
Schematic of a triple extrusion process for HVDC cable production.

**Figure 4:**
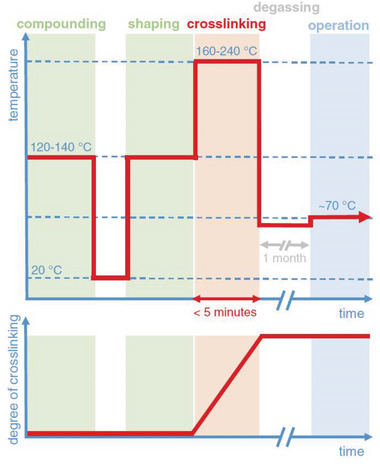
Experienced temperature (top) and the degree of crosslinking of the insulation (bottom) during processing, crosslinking, degassing and operation of an extruded HVDC cable. Image reproduced with permission from ref. [19]. Copyright 2020, Wiley.

**Figure 5:**
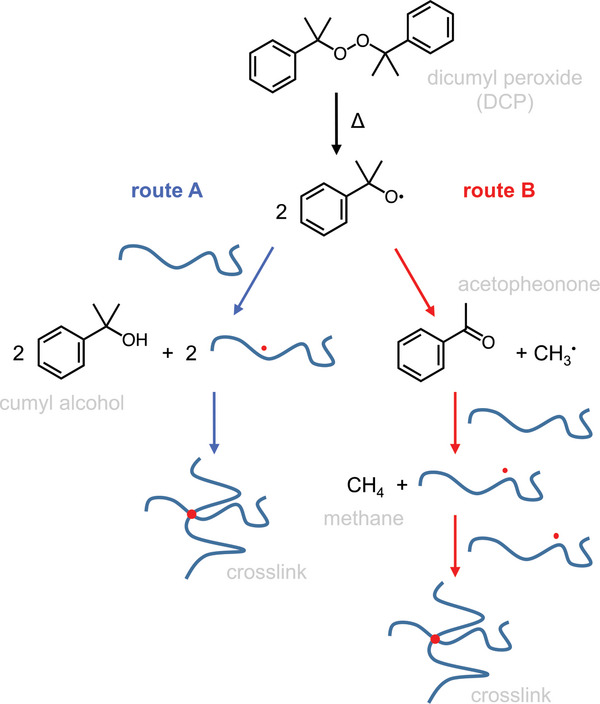
Radical crosslinking of polyethylene with DCP involves decomposition of DCP above 160 °C yielding two radicals that either abstract a hydrogen from a polyethylene chain (route A), producing cumyl alcohol (2‐phenyl‐2‐propanol) as a byproduct (that can further decay into α–methyl styrene and water), or decay into acetophenone and a methyl radical (route B), which then abstracts a hydrogen from a polyethylene chain and forms methane.^[^ ^28^ ^]^

LDPE is widely used as an insulation material because it can be produced with an exceptionally high degree of both physical and chemical cleanliness through high‐pressure polymerization, resulting in a *σ*_*DC*_ as low as 10^−14^ S m^−1^ at 70 °C and an electric field of 30 kV mm^−1^ (Figure 2).^[^ ^18^ ^]^ However, the low onset of melting and relatively low peak $T_{m}^{LDPE}$ ≈110 °C of LDPE compromise the thermomechanical properties of the insulation material at elevated operating temperatures. Therefore, LDPE is commonly crosslinked to ensure that the insulation does not deform at higher temperatures. During cable production, the insulating layer is first applied together with the semicon layers onto the conducting core using a triple extrusion process (**Figure** **3**). During the extrusion process, the melt reaches a temperature of 120 to 140 °C (**Figure** **4**), where LDPE can be safely extruded together with a radical crosslinking agent such as dicumyl peroxide (DCP) without the onset of the curing reaction. During a subsequent vulcanization step, which lasts ≈5 min, the insulation is cured at temperatures above 180 °C (Figure 4), yielding XLPE. At elevated temperatures, the DCP decomposes into two cumyloxy radicals, and these radicals either directly abstract hydrogen from a polyethylene chain, resulting in a free radical on the macromolecule, or first decompose into acetophenone and a methyl radical, followed by hydrogen abstraction (**Figure** **5**). DCP itself is a hazardous substance and the curing process produces a range of harmful and/or volatile byproducts such as water, methane, acetophenone, cumyl alcohol, and α–methyl styrene.^[^ ^28^ ^]^ These byproducts are mobile and high contents can increase the electrical conductivity and breakdown probability of the insulation material,^[^ ^29^, ^30^ ^]^ and therefore must be removed through a degassing step,^[^ ^31^ ^]^ so that a material with a *σ*_*DC*_ comparable to LDPE is obtained, i.e., *σ*_*DC*_ ≈ 10^−14^ S m^−1^ at 70 °C and 30 kV mm^−1^ (Figure 2). This involves the storage of the extruded and cured cable at 50 to 80 °C for several days up to one month, which is time and energy consuming. Since DCP curing is associated with significant shortcomings, there is considerable scope for the development of alternative polyethylene‐based insulation materials with thermomechanical and dielectric properties that are at least on a par with XLPE. In this review, we will discuss alternative avenues, which are currently under investigation, that may allow to either replace the peroxide crosslinking chemistry with a byproduct free (and potentially reversible) curing process or avoid chemical crosslinking altogether.

## 2 Physical and Covalent Networks in Polyethylene‐Based Materials

A polymer network is a highly ramified system in which each part is connected to the ensemble via network points, which can be a physical or chemical crosslink. The IUPAC gold book defines a crosslink as “*a small region in a macromolecule from which at least four chains emanate, and formed by reactions involving sites or groups on existing macromolecules or by interactions between existing macromolecules*”.^[^ ^32^ ^]^

Physical network points are characterized by non‐covalent interactions such as ionic bonds, hydrogen bonds, π‐π stacking, chain entanglements, etc., which can occur between individual chains in amorphous regions or within ordered, crystalline domains. Physical network points are not permanent in the sense that the physical interactions can be removed below the degradation temperature of the polymer. Most physical networks break up when heated and reform upon cooling, which is an essential requirement for self‐healing and primary recycling, i.e., the thermoplastic material can be reshaped several times with the same processing method such as compression molding or extrusion.

**Figure 6:**
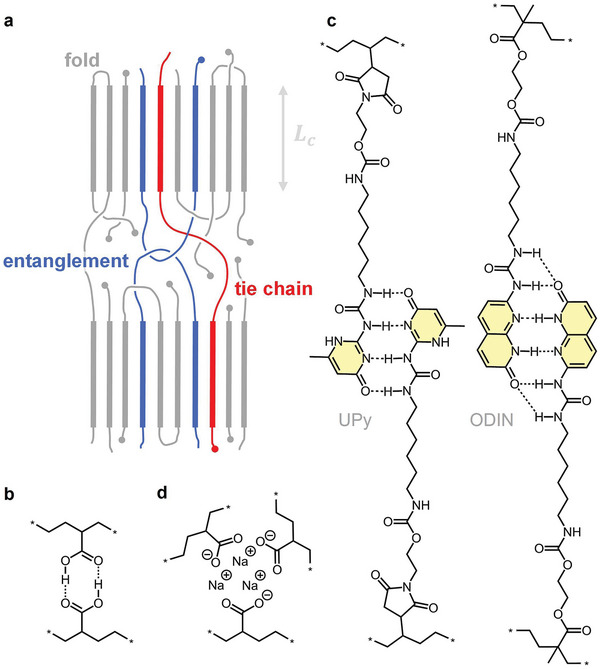
Physical network formation in polyethylene commonly occurs via (a) crystalline lamellae separated by an amorphous phase and connected via trapped entanglements (blue) as well as tie chains (red), which can be augmented by (b) carboxylic acid dimers, (c) dimers of hydrogen‐bonding arrays such as ureidopyrimidinone (UPy) or 1‐(7‐oxo‐7,8‐dihydro‐1,8‐naphthyridin‐2‐yl)urea (ODIN), and (d) sodium carboxylate clusters. 5a reproduced with permission from ref. [34]. Copyright 2016 (CC BY), the Royal Society of Chemistry.

Neat, random polyethylene chains (excluding its semicrystalline structure) only interact via weak dispersion forces. In the melt state, the only network points are entanglements, provided that the molecular weight exceeds the entanglement molecular weight *M_e_* of 1250 g mol^−1^.^[^ ^33^ ^]^ Entanglements are temporary network points because they can dissociate over time through the disentanglement of polymer chains by reptation. In the solid‐state, i.e., below *T_m_*, polyethylene is semi‐crystalline and its nanostructure is characterized by crystalline lamellae that are connected via tie chains and trapped entanglements (**Figure** **6**a).^[^ ^33^ ^]^ A trapped entanglement describes a chain entanglement that is interlocked by adjacent crystallites, which hold the chains in place.

A chemical crosslink generally describes a covalent structure such as a covalent bond between chains that is created upon radical curing with, e.g., DCP (cf. Figure 5). Chemical crosslinking through the formation of covalent bonds leads to an infusible, insoluble polymer network, i.e., a thermoset, which cannot be reshaped by melting and therefore eludes primary recycling. It is, however, feasible to recycle XLPE through, e.g., pyrolysis, resulting in new feedstock for the production of ethylene, through decrosslinking during extrusion using supercritical fluids^[^ ^35^, ^36^ ^]^ or ultrasound,^[^ ^37^ ^]^ as well as through grinding of the material followed by the use of the recyclate as a filler for a thermoplastic polyolefin resin.^[^ ^38^, ^39^ ^]^ The extent of the covalent network depends on the number of chemical crosslinks, and can be described by the gel content, i.e., the insoluble fraction that remains after swelling the material for an extended period of time in an organic solvent such as decahydronaphthalene (decalin).^[^ ^40^ ^]^

An emerging discipline within the field of polymer chemistry deals with adaptable networks, which combine the often‐superior chemical, thermomechanical, and dimensional stability of crosslinked thermoset materials with the reprocessability of thermoplastic materials.^[^ ^41^ ^]^ The adaptable network structure implies that crosslinks can reorganize, meaning that the material is recyclable by heating. Adaptable polymer networks can be divided into two sub‐classes, namely covalent adaptable networks (CANs) and supramolecular networks. CANs are made up of reactive covalent bonds, while supramolecular networks make use of non‐covalent interactions that provide crosslinking sites (cf. hydrogen bonding motifs).

Two types of CANs are recognized that are based on either an associative or dissociative bond exchange mechanism.^[^ ^42^ ^]^ In case of associative CANs breakage of an existing bond, e.g. through transesterification (see Section 5), is accompanied by the formation of a new bond, and hence the network integrity is kept intact due to a fixed crosslink density. This type of exchange behavior is reminiscent of glasses, and thus associative CANs are also referred to as vitrimers.^[^ ^43^ ^]^ To date, only few examples of polyethylene‐based materials with associative network points have been reported, such as graft‐modified HDPE with bis‐dioxaborolane crosslinks^[^ ^44^, ^45^ ^]^ as well as various polyethylene‐based copolymers that were connected via, e.g., thermally reversible crosslinks comprising esters (that can undergo transesterification in the presence of zinc acetate; cf. Section 5),^[^ ^46^ ^]^ vinylogous urethanes^[^ ^47^ ^]^ or silyl ethers.^[^ ^48^ ^]^ In the latter two cases crosslinking could be achieved via reactive extrusion and the exchange mechanism was activated by a temperature increase without the need of any catalyst.

Instead, in case of dissociative CANs, crosslinks break, e.g. through heating, resulting in a loss of network integrity. Subsequently, crosslinks associate again upon cooling. A typical example is crosslinks based on Diels‐Alder adducts where a conjugated diene, e.g. furane, and a substituted alkene, e.g. maleimide, undergo a cycloaddition reaction, resulting in the thermally reversible formation of an adduct. Only few examples of polyethylene‐based dissociative CANs have been reported such as materials with furan/maleimide adducts^[^ ^49^ ^]^ and sulfur bridges^[^ ^50^ ^]^ as dynamic covalent crosslinks. If only the network characteristics and not the type of crosslinks are taken into account, supramolecular networks can be considered as dissociative adaptable networks.

Polyethylene‐based supramolecular networks are composed of copolymers whose comonomers don functional groups that facilitate non‐covalent interactions. A classic example is the incorporation of acrylic acid comonomers into the polyethylene chain. The acid form of ethylene‐acrylic acid copolymers forms carboxylic acid dimers through hydrogen bonding (Figure 6b). Further, the design of dissociative non‐covalent polyethylene networks based on self‐dimerizing multiple hydrogen‐bonding arrays such as ureidopyrimidinone (UPy) and 1‐(7‐oxo‐7,8‐dihydro‐1,8‐naphthyridin‐2‐yl)urea (ODIN) has recently been explored (Figure 6c).^[^ ^51^, ^52^ ^]^

Another type of polyethylene‐based supramolecular networks are ionomers that typically contain a few mole percent of ionic groups.^[^ ^53^ ^]^ Polyethylene based ionomers can be prepared by copolymerization of ethene with an ionic comonomer or by post‐functionalization, for example by grafting reactions during reactive extrusion. The most well‐known polyethylene‐based ionomers are obtained by neutralization of ethylene‐acrylic acid or ethylene‐methacrylic acid copolymers with mono‐ or divalent metal salts comprising Na^+^ or Zn^2+^. Neutralization results in the formation of carboxylate groups that form clusters (Figure 6d), which act as strong network points held together by ionic interactions.

**Figure 7:**
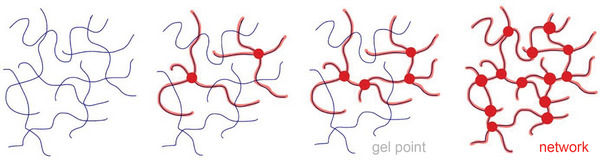
An increase in the number of crosslinked polymer chains (red) turns a polyethylene melt into a continuous network once the gel point is reached.

The properties of a polymeric material are not only determined by the types of crosslinks, but also by the crosslink density. For instance, the plateau modulus of a rubber material scales with the molecular weight between crosslinks *M_c_* according to:

(2) $$M_{c}=\frac{\rho RT}{G^{\prime }}\;$$

where *ρ*, *R*, *T*, and *G*′ are the density, universal gas constant, temperature, and storage modulus, respectively. Prior to a curing reaction such as radical crosslinking of polyethylene with DCP, the only type of network points that are present (in the melt state) are entanglements. Heating leads to the formation of covalent network points that connect polyethylene chains. Once a continuous network that spans the entire system has formed, the so‐called gel point is reached (**Figure** **7**). This is typically accompanied by a sudden increase in melt viscosity.

We now use the above introduced terminology to compare the network points that are present in insulation‐grade LDPE and XLPE. A typical type of LDPE grade that is used for research purposes features ≈2 long‐chain branches per 1 000 carbons, has a crystallinity of ≈50% and features a $T_{m}^{LDPE}$ ≈110 °C.^[^ ^18^ ^]^ In the solid state LDPE contains different types of physical network points: crystallites, entanglements and trapped entanglements, of which only entanglements remain above $T_{m}^{LDPE}$. Upon curing with DCP, covalent C**–**C crosslinks are added. XLPE used for research purposes has a similar crystallinity as the LDPE from which it is produced since it typically features only ≈2 to 3 crosslinks per 1 000 carbons (gel content typically ≈70%).^[^ ^20^, ^25^ ^]^ The similar crystallinity implies that crosslinking does not impair the storage modulus of the insulation material below $T_{m}^{LDPE}$.^[^ ^20^, ^24^ ^]^ The covalent crosslinks persist above $T_{m}^{LDPE}$ where they interlock (some) entanglements, and hence turn them into permanent network points. Hence, above a certain crosslink density, the cured material no longer flows above $T_{m}^{LDPE}$, which prevents primary recycling.

Different strategies involving chemical and/or physical crosslinks are being developed as byproduct‐free and/or recyclable alternatives to DCP‐cured LDPE. These will be discussed in the following sections. Similar to traditional XLPE, it is important that the number of crosslinks is kept low in order to not unduly impede crystallization, which would compromise the mechanical and electrical properties of the base resin. In particular, we will cover (1) how click chemistry type reactions can be used to form thermoset‐type covalent networks without the release of byproducts, (2) how higher‐melting polymers can be used to introduce a physical network and thus design thermoplastic insulation materials, and (3) how adaptable networks can be used to create thermoset‐like materials that can be reprocessed similar to thermoplastic materials.

## 3 Click Chemistry Type Crosslinking of Polyethylene Copolymers

Crosslinkable polyolefins can be realized through the incorporation of functional comonomers that react on demand. One example is silane curing, which involves the formation of covalent bonds between vinyltrimethoxysilane comonomers upon initiation with water,^[^ ^54^ ^]^ which however is not suitable for a high‐voltage insulation material because the presence of water might introduce additional charge carriers.^[^ ^55^ ^]^

**Figure 8:**
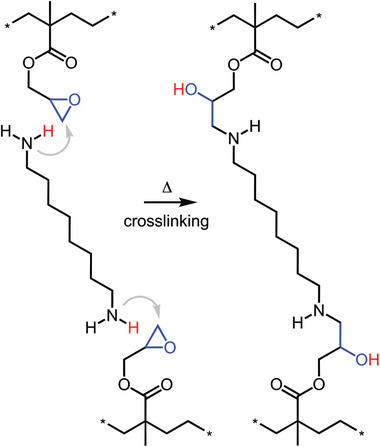
Crosslinking of p(E‐*stat*‐GMA) with 1,8‐diaminooctane (DAO).

An interesting alternative are crosslinking concepts based on click chemistry type reactions, a term that we here use to describe high‐yield curing reactions that generate no byproducts. Suitable formulations are those that comprise ethylene‐glycidyl methacrylate copolymers, p(E‐*stat*‐GMA), which are widely used for reactive compatibilization of polymer blends. The comonomer features an epoxy functional group that can react with a variety of moieties such as amines, phenols, thiols, hydrazides, isocyanates or acids without the release of any byproducts.^[^ ^56^ ^]^ Alternatively, copolymers that incorporate maleic anhydride or maleimide comonomers can be used, which must first be activated with, e.g., H_2_O before they can react with an epoxy group.^[^ ^57^ ^]^ To obtain a polymer network the crosslinking agent should carry at least two functional groups. Curing of p(E‐*stat*‐GMA) with 1,8‐diaminooctane (DAO) (**Figure** **8**) led to a high gel content of more than 80% after 10 min at 200 °C,^[^ ^56^ ^]^ which however is too slow for a cable extrusion process (cf. Figure 4; the curing step preferably takes less than 5 min). The curing kinetics with DAO or the bisphenol curing agent of 2,2‐bis(4‐hydroxy‐3‐methylphenyl) propane (BPP) could be substantially improved by using a titanium‐based Lewis acid, reaching a gel content of 90% after curing for 5 min at 220 °C with BPP.^[^ ^58^ ^]^ The high curing rate could be explained with the in situ generation of a new titanium‐phenoxide catalyst, which colored the cured copolymer orange. While aesthetically pleasing, a non‐white insulation material is undesirable since it would complicate quality control during cable production, which entails the inspection of cable sections submerged in an at least 110 °C hot silicon oil bath (i.e., $T>T_{m}^{LDPE}$ so that the insulation becomes transparent) where any contaminants and protrusions become apparent. Moreover, discoloration is a powerful indicator for thermo‐oxidation of the polymer melt in the extruder.

While promising in terms of curing rate, catalysts and unreacted low molecular‐weight crosslinking agents that are present in the cured insulation material may act as additional charge carriers, which would increase the electrical conductivity and the probability for breakdown at high electric fields. Accordingly, p(E‐*stat*‐GMA) cured with low molecular‐weight amine and phenol crosslinking agents (i.e., BPP or DAO) showed an about one order of magnitude higher DC electrical conductivity $\sigma_{DC}^{BDS}$ than XLPE (cf. **Table** **1**),^[^ ^26^ ^]^ which may arise due to the presence of unreacted crosslinking agent.

**Table 1:** DC electrical conductivity at 70 °C of LDPE, XLPE, and PE copolymer formulations comprising p(E‐*stat*‐GMA) with a glycidyl methacrylate content of 8 wt.% cured with DAO, BPP + Ti(2‐EtHexO)_4_ catalyst or p(E‐*stat*‐AA), obtained using BDS at 10 V mm^−1^, $\sigma_{DC}^{BDS}$, or leakage current measurements at 20 kV mm^−1^, $\sigma_{DC}^{HV}$.^[^ ^19^, ^61^ ^]^ Leakage current measurements at quasi steady state are more relevant for HVDC insulation materials because a high electric field is applied but nevertheless good agreement with BDS is here observed.

material	$\sigma_{DC}^{BDS}$ (10^−14^ S m^−1^)	$\sigma_{DC}^{HV}$ (10^−14^ S m^−1^)
LDPE	1.8	1.5
XLPE	2.2	2.3
p(E‐*stat*‐GMA) + DAO	25	–
p(E‐*stat*‐GMA) + BPP + Ti catalyst	12	–
1.7:1 p(E‐*stat*‐GMA):p(E‐*stat*‐AA)	2.1	0.9
1.7:1:2.7 p(E‐*stat*‐GMA):p(E‐*stat*‐AA):LDPE	2.8	1.6

**Figure 9:**
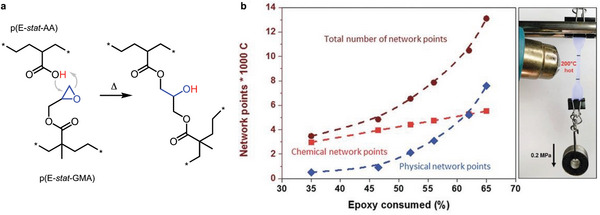
(a) Byproduct‐free crosslinking of an ethylene–acrylic acid copolymer p(E‐*stat‐*AA) and an ethylene–glycidyl methacrylate copolymer p(E‐*stat*‐GMA), and (b) chemical and physical network points (e.g., trapped entanglements) per 1 000 carbon atoms as a function of the percentage of epoxy groups consumed by the crosslinking reaction (left), which result in a high degree of creep resistance during heating to ≈200 °C (right). Figure reproduced with permission from ref. [25]. Copyright 2018 (CC BY‐NC), the Royal Society of Chemistry.

An interesting alternative is crosslinking of a blend of two polyethylene copolymers, p(E‐*stat*‐GMA) and a statistical ethylene‐acrylic acid copolymer, p(E‐*stat*‐AA), which does not involve any low molecular‐weight crosslinking agents that may compromise the electrical conductivity (**Figure** **9**a). The uncatalyzed copolymer blend could be safely extruded at 120 to 140 °C, while subsequent heating to above 170 °C triggered curing through a reaction between epoxy and carboxyl groups, reaching a gel content of 95% after 5 min at 200 °C that bestows the thermoset with a high degree of creep resistance at temperatures above the melting temperature of polyethylene (Figure 9b). The high gel content of cured copolymer blends arises because each reaction not only results in one covalent crosslink, but also leads to trapped entanglements, the number of which increases as the curing reaction proceeds (Figure 9b). It is necessary to avoid premature curing during extrusion, which, besides the selection of copolymers with a sufficiently low comonomer content, can be readily achieved through the addition of neat LDPE to the copolymer blend, thus creating a ternary blend.^[^ ^19^ ^]^

The use of polyethylene copolymer blends facilitates the design of formulations that can be cured without generating byproducts, which can be anticipated to result in a crosslinked insulation material with advantageous dielectric properties. Both broadband dielectric spectroscopy (BDS) at an electric field of 10 V mm^−1^ and leakage current measurements at high electric fields of 10–50 kV mm^−1^ indicate that cured blends of p(E‐*stat*‐GMA) and p(E‐*stat*‐AA) feature a very low electrical conductivity that is on a par with values measured for both ultra‐clean LDPE and a commercial XLPE grade, i.e., $\sigma_{DC}^{HV}$ ≈ 10^−14^ S m^−1^ at 70 °C and 20 kV mm^−1^ (Figure 2 and Table 1).^[^ ^19^, ^59^ ^]^ Not all carboxyl and epoxy functional groups react, which indicates that neither hydrogen bonding (cf. carboxylic acid dimers) nor the presence of unreacted polar comonomers compromises the dielectric properties.^[^ ^25^ ^]^ Importantly, cured blends of p(E‐*stat*‐GMA) and p(E‐*stat*‐AA) feature invariant thermomechanical and dielectric properties ($\sigma_{DC}^{HV}$ ≈3·10^−14^ S m^−1^ at 70 °C and 20 kV mm^−1^; similar tan δ) upon aging at 90 °C in air for at least 100 days, provided that a suitable antioxidant such as pentaerythritol tetrakis[3‐[3,5‐di‐tert‐butyl‐4‐hydroxyphenyl]propionate (Irganox 1010) is added.^[^ ^60^ ^]^ Moreover, cured polyethylene copolymer blends feature a low dielectric loss tangent tan δ at 70 °C and, e.g., 50 Hz with values that are comparable to those measured for LDPE and XLPE,^[^ ^59^ ^]^ indicating that the introduction of polar comonomers does not result in additional dielectric losses. Evidently, click chemistry type crosslinked polyethylene copolymer blends display a promising combination of thermomechanical and dielectric properties.

## 4 Thermoplastic Polymer Blends

Thermoplastic insulation materials receive increasing attention due to environmental concerns related to DCP curing but also to simplify cable manufacturing.^[^ ^62^, ^63^ ^]^ Unlike chemically crosslinked LDPE, thermoplastic materials can be re‐processed from the melt through extrusion, which facilitates primary mechanical recycling. A suitable thermoplastic insulation material should (1) display a high degree of form stability comparable to XLPE up to a temperature of at least $T_{m}^{LDPE}$ ≈110 °C, and ideally also at $T>T_{m}^{LDPE}$, and (2) allow reshaping at higher temperatures. While these criteria are fulfilled by polyolefins such as high‐density polyethylene (HDPE) or isotactic polypropylene (iPP), which have a higher melting temperature than LDPE, these materials alone cannot simultaneously match the outstanding melt flow properties, low‐temperature flexibility and the high degree of cleanliness –advantageous for maintaining a low electrical conductivity and high dielectric strength– that can be achieved with LDPE. Therefore, blends or copolymers, which combine different polyolefins and hence display the desired portfolio in terms of both thermomechanical and dielectric properties, currently receive considerable attention (see Figure 2).

**Figure 10:**
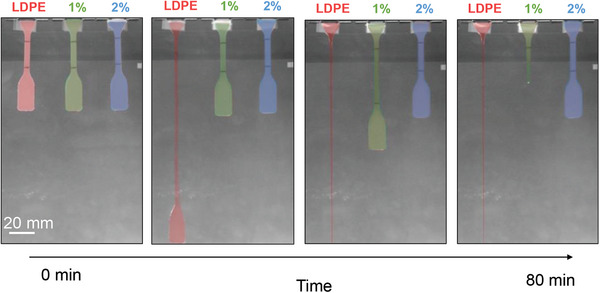
Creep elongation of neat LDPE (red) and LDPE:HDPE blend samples containing 1 wt.% (green) or 2 wt.% HDPE (blue) under their own weight over 80 min at ≈117 °C. Figure adapted with permission from ref. [64].  Copyright 2017, Wiley.

A number of studies have explored the use of blends of HDPE and LDPE as insulation materials.^[^ ^18^, ^64^, ^65^, ^66^, ^67^, ^68^ ^]^ HDPE has a $T_{m}^{HDPE}$ ≈135 ˚C and a narrower melting interval than LDPE, resulting in a higher heat deflection temperature. HDPE and LDPE are partially or fully miscible in the melt, depending on, e.g., the molecular weight of the polymers^[^ ^69^ ^]^ as well as the degree of branching of LDPE,^[^ ^70^, ^71^ ^]^ which determines the solid‐state microstructure that can develop during solidification. Cooling of a phase‐separated melt will give rise to a coarse solid‐state microstructure characterized by large domains that are rich in either polymer. Instead, a homogeneous melt facilitates the formation of a fine nanostructure where, depending on the cooling rate, crystals of either polymer as well as co‐crystals^[^ ^72^, ^73^ ^]^ are finely dispersed.^[^ ^64^ ^]^ The selection of an HDPE grade with a sufficiently high molecular weight can yield a nanostructure that features HDPE crystallites and HDPE:LDPE co‐crystals connected via tie chains and trapped entanglements, which hence form a network that traverses the entire insulation material.^[^ ^65^ ^]^ Between the melting temperature of LDPE and HDPE this network of higher‐melting crystallites is able to arrest creep, resulting in improved form stability across the temperature window $T_{m}^{LDPE}<T<T_{m}^{HDPE}$ as compared with neat LDPE, which is already completely molten.^[^ ^65^ ^]^ HDPE can, in fact, be used as an additive for LDPE since, depending on the choice of resin, only 1–2 wt.% HDPE is needed to arrest creep of the LDPE matrix above $T_{m}^{LDPE}$ (**Figure** **10**).^[^ ^64^ ^]^

The dielectric properties of HDPE:LDPE blends depend on the crystal size and distribution, which can be optimized by tuning both the blend composition as well as processing parameters.^[^ ^16^, ^18^ ^]^ The addition of small amounts of HDPE to LDPE can significantly reduce the DC electrical conductivity.^[^ ^18^, ^27^ ^]^ Blends containing as little as 1–2 wt.% HDPE feature a $\sigma_{DC}^{HV}$ ≈10^−15^ S m^−1^ at 70 °C and 30 kV mm^−1^ (Figure 2), which is approximately one order of magnitude lower than values reported for neat LDPE and XLPE.^[^ ^18^ ^]^ The reduction in electrical conductivity can be attributed to a reduction in the mobility of charge carriers through the presence of thicker HDPE crystalline lamellae, which according to Montanari et al. feature a higher density and depth of trap sites.^[^ ^74^ ^]^ A further reduction in $\sigma_{DC}^{HV}$ can be achieved through the addition of a conductivity‐reducing additive such as octyl‐silane‐coated Al_2_O_3_ nanoparticles to LDPE:HDPE binary blends, which suggests that the nanoparticles and the higher‐melting polyolefin have a synergistic conductivity‐reducing effect.^[^ ^27^ ^]^

Polypropylene (PP) based insulation materials are attractive because of the high $T_{m}^{PP}$ of up to 170 °C, which can impart a high degree of form stability considerably above $T_{m}^{LDPE}$. Isotactic PP (iPP), which is widely used for capacitor and supercapacitor applications,^[^ ^75^ ^]^ tends to feature a very low electrical conductivity of *σ*_*DC*_ ≤ 10^−15^ S m^−1^ at 70 °C (Figure 2),^[^ ^76^ ^]^ which can be further reduced through the use of β‐nucleating agents^[^ ^77^ ^]^ as well as conductivity‐reducing additives such as metal oxide nanoparticles^[^ ^78^, ^79^, ^80^ ^]^ or organic semiconductors such as C_60_.^[^ ^12^ ^]^ However, on its own isotactic PP is not suitable as an HVDC insulation material because of its high glass transition temperature $T_{g}^{PP}$ ≈ 0 °C, high stiffness and low *κ* ≈ 0.26 W m^−1^ K^−1^ at 70 °C (note that *κ* of iPP depends on the microstructure and can range from 0.1 to 0.3 W m^−1^ K^−1^),^[^ ^21^ ^]^ which complicates heat management of cables (cf. Equation 1). Two broad strategies have been explored to create suitable PP‐containing insulation materials that (1) first consider PP and then aim to reduce the stiffness through the incorporation of a rubbery phase, or (2) first consider LDPE, which is then reinforced through the introduction of a PP‐rich phase. In both cases, blending or copolymerization are used to modify the thermomechanical properties of the reference material, i.e., iPP or LDPE.

The use of PP‐inspired formulations has been summarized by several recent reviews.^[^ ^63^, ^75^, ^81^, ^82^ ^]^ First cables with a thermoplastic insulation are now available commercially,^[^ ^83^ ^]^ which besides propylene‐based materials also contains a dielectric fluid based on mineral oil.^[^ ^82^ ^]^ Propylene‐based materials that have been explored include random^[^ ^84^, ^85^, ^86^ ^]^ and blocky propylene‐ethylene copolymers^[^ ^85^, ^86^ ^]^ as well as heterophasic and random heterophasic propylene‐ethylene copolymers,^[^ ^84^, ^87^, ^88^ ^]^ i.e., a PP homo‐ or copolymer matrix with ethylene‐propylene rubber (EPR) inclusions prepared by a sequential polymerization process.^[^ ^89^ ^]^ The incorporation of as little as 2% ethylene comonomer into the polypropylene backbone reduces the stiffness at room temperature and results in a tough material that can undergo considerable tensile deformation without fracture.^[^ ^84^, ^85^ ^]^ At the same time, a $T_{m}^{PP}$ of 150 to 160 °C is maintained, which ensures that the material displays a relatively high storage modulus at elevated temperatures, e.g. more than 100 MPa at 120 °C in case of propylene‐ethylene copolymers.^[^ ^84^ ^]^ The electrical properties of polypropylene copolymers such as the electrical conductivity and the dielectric strength have been studied extensively (albeit often only at room temperature)^[^ ^84^, ^85^, ^86^, ^87^, ^88^ ^]^ and materials such as random heterophasic propylene‐ethylene copolymers can offer a high $T_{m}^{PP}$ = 150 °C as well as a low $\sigma_{DC}^{HV}$ = 7·10^−15^ S m^−1^ at 70 °C and 30 kV mm^−1^ (Figure 2).^[^ ^26^ ^]^

Moreover, a wide range of blends have been considered such as binary blends of (1) iPP with an propylene‐ethylene copolymer,^[^ ^68^, ^90^, ^91^ ^]^ an ethylene‐octene copolymer^[^ ^91^, ^92^, ^93^ ^]^ or a propylene‐butylene copolymer,^[^ ^90^ ^]^ (2) ternary blends of iPP with an ethylene‐octene as well as a propylene‐ethylene copolymer,^[^ ^23^, ^94^, ^95^ ^]^ and (3) binary blends of a propylene‐ethylene copolymer with a polystyrene‐*b*‐(ethylene‐*co*‐butylene)‐*b*‐polystyrene (SEBS) elastomer.^[^ ^96^ ^]^ Some of the most promising blends comprise iPP and ethylene‐propylene copolymers.^[^ ^68^, ^90^, ^91^ ^]^ For example, a 50:50 blend of iPP and an ethylene‐propylene copolymer with 9 mol% ethylene content is as flexible as XLPE at low temperatures and at the same time features a considerable storage modulus of more than 10 MPa at 120 °C.^[^ ^68^ ^]^ Another example is a ternary blend of iPP with an ethylene‐octene copolymer and a PP copolymer (with EPR inclusions) as a third component, resulting in ternary blends with a storage modulus of 10 MPa at 150 °C (thanks to the high $T_{m}^{PP}$ = 166 °C) and $\sigma_{DC}^{HV}$ ≈ 10^−15^ S m^−1^ at 80 °C and ≈8 kV mm^−1^ (Figure 2).^[^ ^23^ ^]^

**Figure 11:**
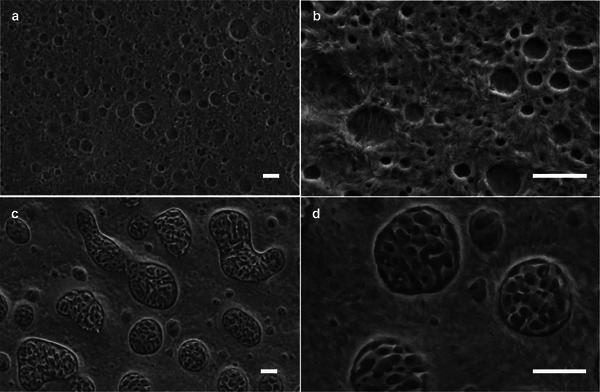
Scanning electron microscopy (SEM) images of cryofractured and etched samples of (a, b) a 25:75 iPP:LDPE binary blend, and (d,e) a 10:90 SEBS:(iPP:LDPE)_25:75_ ternary blend (all scale bars correspond to 2 µm). Images reproduced with permission from ref. [97]. Copyright 2021 (CC BY), Wiley.

The second strategy is based on blends of PE and iPP with the goal to combine the superior processability of LDPE with the higher form stability of iPP at elevated temperatures. Compounding of these two immiscible polymers will create a phase‐separated microstructure with weak domain interfaces (**Figure** **11**), which is why binary blends are seldom considered.^[^ ^82^ ^]^ Uncompatibilized iPP:LDPE blends only display a high elastic modulus and creep resistance above $T_{m}^{LDPE}$ if the iPP phase is continuous,^[^ ^97^ ^]^ which requires that^[^ ^98^ ^]^:

(3) $$\frac{\eta_{LDPE}}{\eta_{PP}}\cdot \frac{\phi_{PP}}{\phi_{LDPE}}>1$$

where *η*_*LDPE*_ and *η*_*PP*_ are the melt viscosities of LDPE and iPP and *ϕ*_*LDPE*_ and *ϕ*_*PP*_ are their volume fractions. Hence, the melt flow properties of the two materials determine how much iPP must be added to LDPE to realize a significant reinforcing effect above $T_{m}^{LDPE}$. Blends with a sufficiently high iPP content, and hence a continuous iPP phase, can display a storage modulus on the order of 100 MPa at 120 °C.^[^ ^21^, ^97^ ^]^ Gratifyingly, iPP:LDPE binary blends display a $\sigma_{DC}^{HV}$ in between values of 3⋅10^−14^ and 10^−15^ S m^−1^ measured at 70 °C and 30 kV mm^−1^ for neat LDPE and iPP, respectively,^[^ ^21^ ^]^ which suggests that judicious blending of different polyolefins allows to tune both the thermomechanical and dielectric properties.

The microstructure and hence the thermomechanical properties of iPP:LDPE blends can be modified through the addition of additives that function as a compatibilizer or at least modify the interface between phase‐separated domains. The addition of a small amount of SEBS, which is widely used as an impact modifier for PP, can improve the thermomechanical properties of iPP:LDPE blends even if the iPP content is too low to result in continuous iPP domains.^[^ ^21^, ^97^ ^]^ The SEBS additive assembles at the interface between isolated iPP domains and the LDPE matrix (Figure 11) and improves the stress transfer above $T_{m}^{LDPE}$.^[^ ^97^ ^]^ Such SEBS:iPP:LDPE ternary blends resist melt creep at 130 °C at a moderate load of 1 kPa, which is comparable to the compressive stress exerted by a conducting aluminum core in a power cable. The addition of SEBS results in ternary blends with promising thermomechanical and dielectric properties superior to XLPE, e.g. a storage modulus of 10 MPa at 150 °C and $\sigma_{DC}^{HV}$ ≈ 2⋅10^−15^ S⋅m^−1^ at 70 °C and 30 kV⋅mm^−1^ (Figure 2), while maintaining a relatively high *κ* ≈ 0.3 W m^−1^ K^−1^ at 70 °C (**Table** **2**).^[^ ^21^ ^]^ Furthermore, the dielectric properties of SEBS:iPP:LDPE ternary blends can be adjusted through the addition of conductivity‐reducing additives such as octyl‐silane‐coated Al_2_O_3_ nanoparticles, which results in ultra‐low $\sigma_{DC}^{HV}$ values.^[^ ^24^ ^]^

**Table 2:** Storage modulus *E*′ at 150 °C, obtained with dynamic mechanical analysis (DMA) in tensile mode, DC electrical conductivity $\sigma_{DC}^{HV}$ at 70 °C, obtained using leakage current measurements at 30 kV mm^−1^, and thermal conductivity *κ* at 70 °C of LDPE, XLPE, iPP and a 20:38:42 wt.% SEBS:iPP:LDPE ternary blend (compounded at 180 °C).^[^ ^21^ ^]^

material	*E*′ at 150 °C [MPa]	$\sigma_{DC}^{HV}$ at 70 °C [10^−14^ S m^−1^]	κ at 70 °C [W m^−1^ K^−1^]
LDPE	0.02	2.5	0.36
XLPE	0.4	4.0	0.33
iPP	145	0.1	0.26
SEBS:iPP:LDPE	11	0.2	0.30

Other potential compatibilizers for PP and PE include olefinic random, multiblock and graft copolymers,^[^ ^99^, ^100^ ^]^ EPRs and ethylene‐octene copolymers.^[^ ^82^ ^]^ Another approach comprises reactive compounding to create PP‐PE type copolymers in situ during processing. For instance, polypropylene‐graft‐maleic anhydride (PP‐*graft*‐MA) can react with p(E‐*stat*‐GMA) during compounding despite the presence of as much as 70 wt.% LDPE, resulting in an enhanced melt strength above $T_{m}^{LDPE}$ and a DC electrical conductivity on a par with that of LDPE and XLPE.^[^ ^20^ ^]^ The recyclability of the ternary blend was confirmed by re‐extrusion, which did not overly affect the thermomechanical and dielectric properties.

## 5 Adaptable Networks

Polyethylene based adaptable networks are a class of materials that may allow to combine the advantages of thermoset and thermoplastic insulation, e.g. a high elastic modulus above $T_{m}^{PE}$ as well as the possibility to reprocess the material by remelting. Similar to the byproduct free thermoset solutions discussed in Section 3, the functional groups that are needed to form adaptable networks must not deteriorate the dielectric properties. At the same time, the kinetics of dynamic bonds must be sufficiently fast so that the material can be processed via melt extrusion (cf. Figure 3). While a number of polyethylene based adaptable networks have been realized (see Section 2), only few studies have explored their suitability as high‐voltage insulation materials.

**Figure 12:**
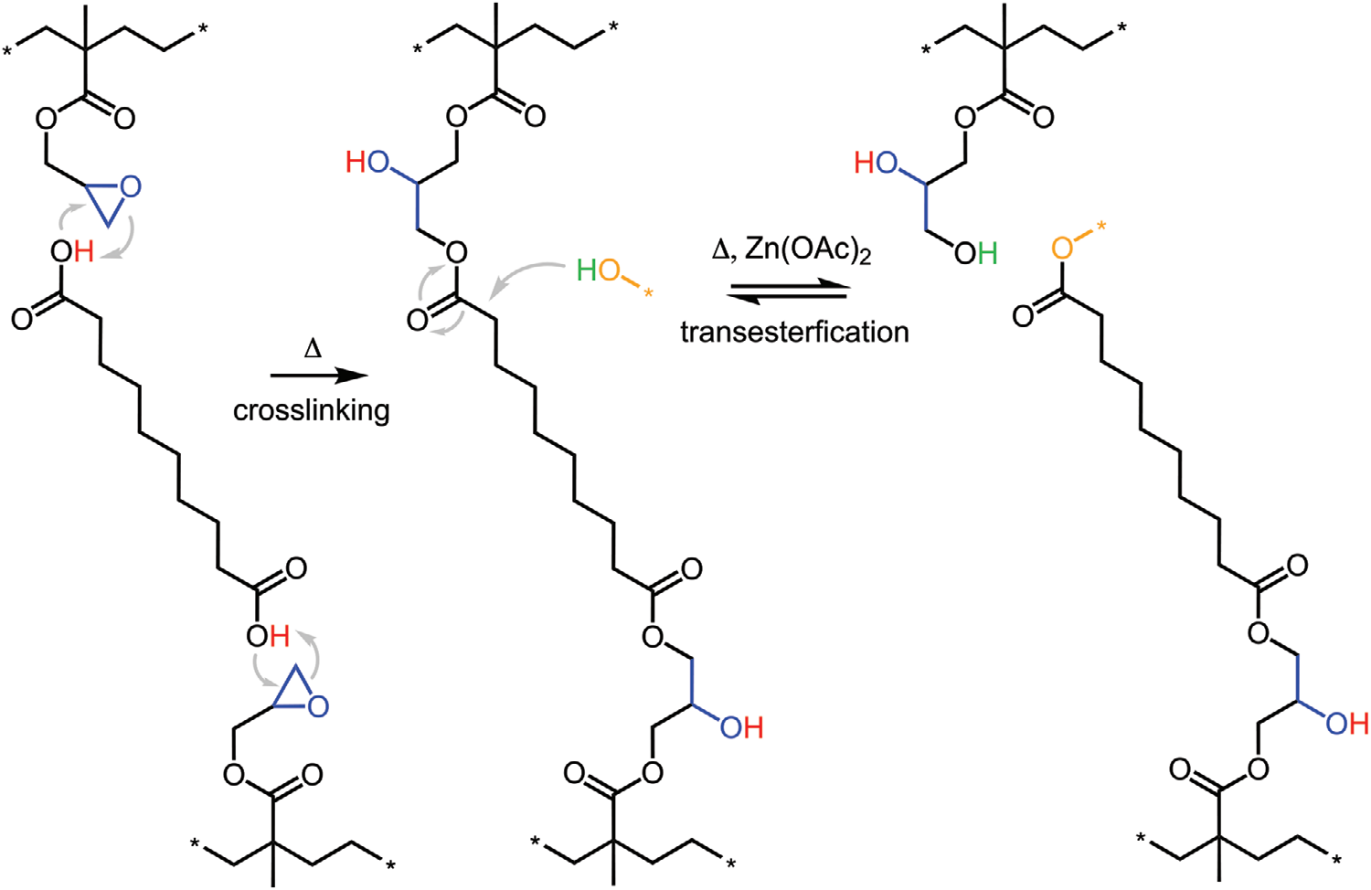
Crosslinking of p(E‐*stat*‐GMA) with 1,8‐octanedicarboxylic acid and transesterification in the presence of zinc acetate, Zn(OAc)_2_.

One type of CAN that has recently been studied utilizes transesterification in crosslinked p(E‐*stat*‐GMA),^[^ ^46^ ^]^ which is an extension of the byproduct‐free curing concept introduced in Section 3. The copolymer p(E‐*stat*‐GMA) was crosslinked by reacting epoxy groups with 1,8‐octanedicarboxylic acid, resulting in the formation of β‐hydroxy ester bonds (**Figure** **12**), which yielded a thermoset with a gel content of up to 80%, a storage modulus of up to 5 MPa at 150 °C and a low electrical conductivity $\sigma_{DC}^{BDS}$ < 10^−14^ S m^−1^ at room temperature. Transesterification in the presence of zinc acetate was triggered by heating to 180 °C leading to gradual stress relaxation above the melting temperature.^[^ ^46^ ^]^ While reprocessing through repeated compression molding could be carried out, the rate of exchange reactions would need to increase significantly to facilitate melt extrusion of the recyclate.

Among supramolecular networks, polyethylene‐based ionomers are interesting candidates for high‐voltage insulation. A commercially available neutralized ethylene‐methacrylic acid copolymer (Surlyn) has been found to feature a surprisingly low $\sigma_{DC}^{HV}$ = 3⋅10^−14^ S m^−1^at 70 °C and 30 kV mm^−1^, which is comparable to XLPE based high‐voltage insulation, but features a too low melting temperature of 90 °C (Figure 2).^[^ ^22^ ^]^ In addition, the presence of cations in this type of ionomer poses the risk of ionic conduction at high electric fields, which complicates their use as a high‐voltage insulation material.

**Figure 13:**
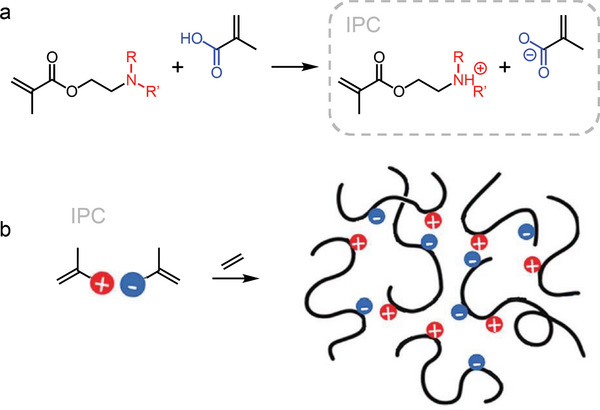
a) Mixing of an amine terminated methacrylate and methacrylic acid results in an ammonium:carboxylate ion pair comonomer (IPC) with R, R’ = methyl, ethyl, tert‐butyl; and b) schematic of free‐radical polymerization of an IPC in the presence of ethene, resulting in an ionomer. Figure adapted with permission from ref. [22].  Copyright 2023 (CC BY‐NC), Wiley.

Recently, a new approach for the synthesis of polyethylene‐based ionomers was introduced that foresees the reaction of different ion pair comonomers (IPCs), composed of methacrylic acid and amine‐terminated methacrylates differing in the type and number of substituents on the amine group (**Figure** **13**).^[^ ^22^ ^]^ IPCs are reacted with ethylene using a high‐pressure/high‐temperature free radical copolymerization method. The resulting type of ionomer contains no by‐products and does not require a neutralization step, thus avoiding possible drawbacks associated with mobile metal ions at high electric fields. These all‐organic ionomers display thermomechanical and dielectric properties that can be tuned by changing the type of amine substituents (Figure 13), yielding a storage modulus of 1 to 3 MPa at 150 °C as well as a low $\sigma_{DC}^{HV}$ = 1 to 6·10^−14^ S m^−1^ at 70 °C and 30 kV mm^−1^ and a *κ* = 0.35 to 0.39 W m^−1^ K^−1^ comparable to XLPE (Figure 2). Strikingly, the presence of IPCs did not increase the dielectric loss tangent tan δ compared to LDPE at, e.g., 0.01 Hz, where DC conduction losses dominate, as well as 50 Hz, the AC frequency used in Europe. Importantly, the ionomers could be melt‐processed, and melt‐pressed films could be redissolved in p‐xylene at 100 °C, which suggests that the material does not form an inseparable network, a prerequisite for reprocessing. Moreover, extensional rheology measurements indicated that tertiary amine‐based ion pairs with sufficiently bulky alkyl groups at the ammonium cation, which increase the anion/cation distance, can reorganize to a greater extent,^[^ ^22^ ^]^ which suggests that the melt processability can be tuned. Hence, it appears that the selection of the right type and number of ionomer type network points may facilitate the design of thermoset‐like insulation materials that can be reprocessed.

## 6 Conclusions and Outlook

Several strategies are emerging that may allow to replace peroxide crosslinking chemistry with either (1) thermoset materials that can be cured similar to XLPE but without a hazardous crosslinking agent and without the release of byproducts, or (2) blend formulations that remain thermoplastic. Both approaches may simplify the production of HVDC cables since the degassing step may become obsolete. In addition, thermoplastic materials have the potential to ease reprocessing of the insulation once a cable has reached the end of its lifetime. Compared with XLPE, polypropylene‐based materials likely offer additional advantages in terms of improved thermomechanical as well as dielectric properties, especially at elevated temperatures, which may allow to increase the power rating of HVDC cables beyond values that are possible today.

Besides alternative curing mechanisms that result in permanent crosslinks, recent work has explored polyethylene‐based materials that feature adaptable network points, thus combining the advantages of thermoset and thermoplastic materials. It can be anticipated that other associative or dissociative crosslinking mechanisms can be borrowed from the field of CANs, resulting in polyethylene‐based insulation materials that are fully reprocessable, i.e., through melt extrusion. A remaining challenge is the relatively low rate of dissociation/association or exchange reactions, which is often limited by the high viscosity of the polymer resin. Adaptable networks that feature long‐term stability at the cable operating temperature but can rapidly reorganize when heated to a higher processing temperature would be needed to facilitate manufacturing at industrial speeds via cable extrusion. Regardless, it may become possible to incorporate self‐healing functionality into the next generation of insulation materials for extruded HVDC cables. The materials discussed in this review exhibit promising thermomechanical and dielectric properties (electrical conductivity; loss tangent) but further measurements should be carried out to investigate, e.g., the effect of long‐term aging, the tendency for space charge accumulation and the dielectric strength.

The development of new concepts for insulation materials should ultimately be followed up by life cycle assessment to identify solutions that are not only attractive from a technology, but also sustainability point of view. Both, (reversible) thermoset and thermoplastic materials have the potential to mature into an attractive technology for future HVDC cable insulation materials.

## Conflict of Interest

The authors declare no conflict of interest.
